# Evaluation of primate anti-pig xenoreactive immune responses using immortalized aortic endothelial cells from genetically modified pigs

**DOI:** 10.3389/fimmu.2026.1936313

**Published:** 2026-09-09

**Authors:** Man Zhang, Hao Feng, Junlong Wu, Jie Lian, Mengting Chen, Yong Wang, Song Chen, Lan Zhu, Shaoping Deng, Hong Wu, Jiaxiang Du, Dengke Pan, Gang Chen

**Affiliations:** 1 Institute of Organ Transplantation, Tongji Hospital, Tongji Medical College, Huazhong University of Science and Technology, Wuhan, China; 2 Key Laboratory of Organ Transplantation, Ministry of Education, Wuhan, China; 3 National Health Commission (NHC) Key Laboratory of Organ Transplantation, Wuhan, China; 4 Key Laboratory of Organ Transplantation, Chinese Academy of Medical Sciences, Wuhan, China; 5 Department of Pathogen Biology, School of Basic Medicine, Tongji Medical College, Huazhong University of Science and Technology, Wuhan, China; 6 Chengdu Clonorgan Biotechnology Co., Ltd., Chengdu, China; 7 Xenotransplantation and Regeneration Key Laboratory of Sichuan Province, Sichuan ClonOrgan Biotechnology Co., Ltd., Neijiang, China; 8 Laboratory of Hepatic AI Translation, Frontiers Science Center for Disease-Related Molecular Network, West China Hospital, Sichuan University, Chengdu, China

**Keywords:** genetically modified pig, iPAECs, xenoantigen, xenogeneic immune responses, xenotransplantation

## Abstract

Porcine vascular endothelial cells are primary targets of humoral and cellular immune responses in pig-to-primate organ xenotransplantation. Although porcine aortic endothelial cells (PAECs) are widely used to evaluate primate anti-pig immune responses, their limited proliferative capacity poses a significant obstacle to repeated and standardized experimentation. To overcome this challenge, we established immortalized PAEC lines (iPAECs) derived from WT, GTKO, and GTKO/CMAHKO/B4GALNT2KO (triple-knockout, TKO) donor pigs using stable transduction of the SV40 large T antigen. All iPAEC lines expanded stably beyond passage 20, maintained high CD31 expression, and preserved genotype-specific xenoantigen profiles. Anti-pig IgM/IgG binding and complement-dependent cytotoxicity (CDC) against these cells were measured with flow cytometry using pooled rhesus monkey sera (n=20) or human sera (n=20). Compared with WT iPAECs, GTKO iPAECs demonstrated significantly reduced IgM/IgG binding and CDC with human and rhesus sera. Additional deletion of Sda and Neu5Gc in TKO iPAECs markedly reduced human serum reactivity, whereas rhesus sera retained relatively high antibody binding and CDC against TKO iPAECs. Notably, the humoral immune responses remained consistent across primary cells and passages P5, P10, and P20, validating the long-term efficacy of iPAECs as an experimental tool. Preformed anti-pig antibodies in 30 rhesus monkeys were further evaluated using TKO iPAECs and TKO peripheral blood mononuclear cells (PBMCs). The results demonstrated a significant correlation between the two target cell types, supporting their interchangeability for humoral immune assessment. Furthermore, xenogeneic MLR assays confirmed that iPAECs could stimulate human CD4^+^ and CD8^+^ T-cell proliferation, especially following porcine IFN-γ–induced upregulation of SLA-I and SLA-II expression. Collectively, these findings establish donor-derived iPAECs as a reliable, reproducible, and genetically defined *in vitro* platform to comprehensively evaluate humoral and cellular xenogeneic immune responses to genetically modified porcine endothelium.

## Introduction

The ongoing severe shortage of human donor organs is a fundamental challenge in clinical transplantation. Recent advances in multiplex gene editing, particularly CRISPR-Cas9, together with novel immunosuppressive regimens, have enabled substantial progress in pig-to-non-human primate (NHP) kidney xenotransplantation, with multiple cases of graft survival exceeding one year (1–4). Additional studies have also demonstrated encouraging stability and reproducibility (5). These advances have provided a critical foundation for the clinical translation of porcine kidney xenotransplantation. Furthermore, hyperacute rejection in pig-to-human xenotransplantation has been successfully prevented (6–9), primarily because preformed natural anti-pig antibodies in human circulation bind weakly to cells from triple-knockout (GTKO/CMAHKO/β4GalNT2KO; TKO) pigs in which the carbohydrate xenoantigens α-Gal, Sda, and Neu5Gc have been eliminated (10, 11). Building on these advances, several gene-edited pig-to-human kidney xenotransplants have been performed in patients with end-stage renal disease, and the grafts have demonstrated satisfactory early renal function and progressively longer recipient survival.

However, achieving stable long-term xenograft survival remains a major challenge (3). Antibody-mediated rejection (AMR), T cell-mediated rejection (TCMR), and thrombotic microangiopathy (TMA) have all been observed early post-transplantation in preclinical pig-to-NHP models and pig-to-human studies (3, 8, 12–15). A clearer understanding of the mechanisms underlying xenoimmune barriers, particularly the humoral and cellular immune responses linked to endothelial injury, remains a critical issue in this field. Although pig-to-NHP xenotransplantation models continue to be an important preclinical platform for studying xenoimmune responses, their long experimental timelines, high cost, and numerous confounding variables necessitate the use of complementary *in vitro* cell-based tools.

The most commonly used cell types include porcine peripheral blood mononuclear cells (PBMCs) and porcine vascular endothelial cells (16–18). Compared with PBMCs, porcine vascular endothelial cells are directly exposed to the recipient circulation after organ transplantation and are among the earliest sites of natural antibody binding, complement activation, and endothelial injury, making them better suited for assessing donor vascular endothelium-related immune risk (3, 12, 18). Previous studies using porcine endothelial cells have covered a broad range of applications, including measurement of IgM and IgG binding and complement-dependent cytotoxicity (CDC) in human or NHP serum (18), identification of novel xenoantigens (19, 20), comparison of donor cell immunogenicity across different gene-editing backgrounds (16, 17), and investigation of immune responses mediated by human NK cells and T cells against porcine cells (21, 22).

Notably, one study of pig-to-brain-dead human recipient kidney xenotransplantation showed that, compared with PBMCs as target cells, dynamic flow crossmatch monitoring using porcine aortic endothelial cells (PAECs) detected changes in anti-PAEC IgG levels that more accurately reflected AMR onset and its response to treatment (12). These results suggest that porcine endothelial cells are valuable for pre-transplant immunological risk assessment and can also serve as an effective tool for post-transplant dynamic monitoring of xenoreactive antibody responses. In summary, porcine vascular endothelial cells serve a dual role in xenotransplantation: as a key target of humoral and cellular immune-mediated injury, and as an indispensable *in vitro* model for studying AMR, TMA, and cellular immune barriers.

Among porcine endothelial cell sources, PAECs are the most widely used due to their accessibility and well-established culture systems (18, 23). However, primary PAECs have a limited *in vitro* lifespan. They typically begin to dedifferentiate after four to five passages, exhibit marked changes in morphology, phenotype, and function, and ultimately enter cellular senescence (24, 25). Furthermore, gene editing of primary cells presents considerable technical challenges. To overcome these limitations, immortalized endothelial cell lines derived from porcine aorta, renal microvasculature, and liver have been established (16, 17, 24) and used in xenotransplantation-related immunological research. Building on this foundation, we isolated PAECs from wild-type (WT), α1, 3-galactosyltransferase gene-knockout (GTKO), and TKO donor pigs and generated corresponding immortalized cell lines (iPAECs). We systematically evaluated the phenotypic stability of these lines across serial passages and their suitability for studies of antibody binding, CDC, and xenogeneic T-cell immune responses to establish a reliable *in vitro* platform applicable to pre-transplant donor-recipient crossmatching, donor cell immunogenicity assessment, and post-transplant immune monitoring, thereby providing a robust cellular tool to support the clinical translation of xenotransplantation.

## Materials and methods

### Isolation and culture of primary PAECs

Primary PAECs were isolated from WT, GTKO, and triple-knockout pigs (GTKO/β4GalNT2KO/CMAHKO; TKO) (Chengdu Clonorgan Biotechnology Co., Ltd, Chengdu, China). General anesthesia was induced with approximately 2 mL of Zoletil. Following endotracheal intubation, anesthesia was maintained with propofol. Under deep anesthesia, the donor pigs were euthanized by intravenous administration of 10% potassium chloride at 1 mL/kg. Death was confirmed before the thoracic aortas were aseptically harvested. The freshly harvested aortas were immediately transferred into sterile, ice-cold PBS for primary PAEC isolation. Briefly, aortic segments were thoroughly rinsed to remove blood by PBS and surrounding connective tissue. The vessels were then inverted to expose the luminal surface and incubated with 0.2% collagenase type I solution (Sigma, USA) at 37 °C for 20–25 min with gentle agitation. Following digestion, endothelial cells were collected by gentle scraping of the luminal surface and transferred into complete endothelial cell medium (ScienCell, USA) supplemented with 10% fetal bovine serum (FBS; Gibco, USA) to terminate the enzymatic reaction. The cell suspension was centrifuged at 300 × g for 5 min; the pellet was resuspended in endothelial cell growth medium, and the cells were seeded into culture dishes. Cells were maintained at 37 °C in a humidified incubator with 5% CO_2_, and this 10% Gibco FBS–containing medium was used for all subsequent culture and expansion.

### Immortalization of PAECs

To generate immortalized PAECs (iPAECs), primary WT, GTKO, and TKO PAECs were transduced with lentivirus encoding SV40 large T antigen (SV40LT). Following infection, cells underwent antibiotic selection and single-cell cloning. Clones were screened based on endothelial morphology, CD31 expression, and genomic validation. Clones exhibiting stable growth and endothelial purity (>90%) were selected and expanded for subsequent use. iPAECs between passages P5 and P20 were used for all experiments unless otherwise specified.

### Sources of rhesus monkey and human sera

Rhesus monkey sera were obtained from 30 outbred male rhesus monkeys (4–10 years old, Hubei Tianqin Biotechnology Corporation and South China Primates Research Center, Hubei, China). Human sera were obtained from 20 healthy volunteers, including men and women aged 22–44 years, with no history of exposure to pig antigens or alloantigens (such as blood transfusions, a previous failed renal allograft, or pregnancy). Monkey or human sera were stored as either single or pooled samples at −80 °C. When required, decomplementation was performed by heat inactivation at 56 °C for 30 min.

### Source of pig PBMCs

Pig PBMCs were obtained from one TKO Bama miniature pig (Chengdu Clonorgan Biotechnology Co., Ltd., Chengdu, China) that shared the same TKO genetic background as the donor pigs used for PAEC isolation. PBMCs were isolated using Ficoll density gradient centrifugation as previously described (26).

### Flow cytometry

#### Phenotypic characterization of iPAECs

iPAECs were stained for the expression of carbohydrate xenoantigens and swine leukocyte antigen (SLA) molecules, including Gal (isolectin BSI-B4), Sda (Dolichos biflorus agglutinin, DBA), Neu5Gc (chicken anti-Neu5Gc monoclonal antibody), SLA class I (APC-labeled anti-human β2-microglobulin, β2M), SLA-DR (purified mouse anti-pig SLA-DR), and SLA-DQ (purified mouse anti-pig SLA-DQ). Alexa Fluor 488–conjugated goat anti-mouse antibody and Alexa Fluor 488–conjugated goat anti-chicken IgY antibody (Thermo Fisher Scientific, USA) were used as the secondary antibodies. Cells were incubated with the lectins or primary antibodies for 30 min at 4 °C in the dark. For the indirectly stained targets, cells were then incubated with the corresponding secondary antibody for a further 30 min at 4 °C in the dark. Unstained cells served as the negative control for the directly conjugated reagents, and secondary-antibody-only staining as the control for the indirectly stained targets.

#### Serum antibody binding assay

Serum IgM and IgG binding to iPAECs was assessed with flow cytometry. Briefly, iPAECs (2 × 10 (5) cells/tube) were incubated with diluted human or rhesus monkey serum to a final concentration of 10%. Bound antibodies were detected using Alexa Fluor 647-conjugated donkey anti-human IgM and Alexa Fluor 488-conjugated goat anti-human IgG (Jackson ImmunoResearch Laboratories, USA). Stained cells were analyzed on a BD FACSAria flow cytometer (BD Biosciences, San Jose, CA, USA), and the resulting data were processed using FlowJo software (Tree Star, San Carlos, CA, USA). Results were expressed as geometric mean fluorescence intensity (G. Mean).

### Complement-dependent cytotoxicity assay

iPAECs (5×10 (5) cells in 250 µL FACS buffer) were incubated with 50µL heat-inactivated human or rhesus monkey serum at 4 °C for 1 h. After washing with phosphate-buffered saline (PBS), FACS buffer (200 µL) and rabbit complement (50 µL, Cedarlane, Hornby, CA, USA) were added (final concentration 20%), and the samples were incubated at 37 °C for 30 min. The cells were subsequently washed with PBS, stained with propidium iodide (PI) in the dark at 4 °C for 15 min, and finally, 200 µL of FACS buffer was added. Flow cytometry was performed using a BD FACSCelesta (BD Biosciences).

Cytotoxicity was calculated, as follows:% cytotoxicity = ([A−C]/[B−C]) ×100.

where A is the percentage of PI-positive (dead) iPAECs in the test sample, B is the maximal percentage of dead cells obtained by 70% ethanol treatment, and C is the background cell death in medium-only controls.

### Transcriptome profiling of iPAECs

Total RNA was extracted from WT, GTKO, and TKO iPAECs at passages P7, P12, and P16, and the samples underwent quality assessment before library preparation. RNA integrity was evaluated using an Agilent 2100 Bioanalyzer and Qubit Fluorometer. Only samples with an RNA integrity number (RIN) > 7.0 and a 28S:18S ratio > 1.8 were used for library preparation. RNA-seq libraries were constructed using the HieffNGS mRNA Isolation Master Kit for Illumina (Yeasen Biotechnology, Shanghai, China; Cat# 12603). The enriched mRNA was fragmented, followed by first- and second-strand cDNA synthesis, end repair, A-tailing, adapter ligation, size selection, and PCR amplification. The resulting libraries were quantified and subjected to quality assessment before sequencing on an Illumina NovaSeq 6000 platform to generate paired-end 150-bp reads. Sequencing quality was assessed using FastQC (v0.11.5), and low-quality reads were filtered using fastp (v0.21.0). Clean reads were aligned to the *Sus scrofa* reference genome (Sscrofa11.1) using HISAT2 (v2.1.0) with default parameters. Gene expression levels were quantified using StringTie (v2.1.5). Heatmaps were generated based on normalized expression values to visualize the expression patterns of selected genes.

### Xenogeneic mixed lymphocyte reaction

CFSE-labeled human PBMCs (2 × 10 (5)/well) were co-cultured with irradiated (36 Gy) iPAECs as stimulators (at stimulator-responder ratios of 1:1 or 1:5) in 96-well U- bottomed microtiter plates at 37 °C and 5% CO_2_ for six days. For the porcine interferon-γ (pIFN-γ) stimulation experiments, iPAECs were treated with pIFN-γ (50 ng/mL) for 48 hours before being used as stimulators. Proliferating T cells were analyzed by flow cytometry after staining with antibodies against human CD3, CD4, and CD8 on day six after stimulation. The CFSE signal of gated cells was detected by using FACSCelesta flow cytometer (BD Biosciences). Proliferation of human PBMC subpopulations was quantified by CFSE dilution (% CFSE cells).

### Antibodies and reagents

Recombinant porcine interferon-γ (pIFN-γ) was obtained from R&D Systems (Cat# 985-PI; Minneapolis, MN, USA), reconstituted in PBS as a stock solution, and stored at -80 °C until used. RPMI 1640 and fetal bovine serum (FBS) were purchased from Gibco (Grand Island, NY, USA). Carboxyfluorescein diacetate succinimidyl ester (CFSE) was obtained from Thermofisher Scientific.

Antibodies used for flow cytometry included anti-human CD3 (BV650; Cat# 563916), anti-human CD4 (BV510; Cat# 317444), anti-human CD8 (APC-Cy7; Cat# 344714), purified chicken anti-Neu5Gc antibody (Cat# 146903), and anti-human β2-microglobulin (β2M; APC; Cat# 395706) from BioLegend (San Diego, CA, USA); purified mouse anti-pig SLA class II DQ (Cat# MCA1335GA) and purified mouse anti-pig SLA class II DR (Cat# MCA2314GA) from Bio-Rad (Hercules, CA, USA); Alexa Fluor 488–conjugated goat anti-mouse IgG (Cat# A-11029) and goat anti-chicken IgY (Cat# A-11039) from Thermo Fisher Scientific; FITC-conjugated anti-Gal (Isolectin B4; Cat# ALX-650-001F-MC05) from Enzo Life Sciences (Farmingdale, NY, USA); and FITC-conjugated Dolichos biflorus agglutinin (DBA; Cat# FL-1031) from Vector Laboratories (Burlingame, CA, USA). Alexa Fluor 647–conjugated donkey anti-human IgM (Cat# 109-605-043) and goat anti-human IgG (Cat# 109-545-003) were purchased from Jackson ImmunoResearch Laboratories (West Grove, PA, USA).

### Statistical analysis

Significant differences between two groups were determined using the unpaired Student’s t-test. Comparisons among multiple groups were performed using one-way analysis of variance (ANOVA) followed by Tukey’s multiple comparisons test. All statistical analyses were performed using GraphPad Prism version 8 software (GraphPad Software, San Diego, CA, USA). Data were presented as means ± standard error of the mean (SEM). Correlations between PBMC- and iPAEC-based assays were assessed using Spearman’s rank correlation. *p<0.05* was considered statistically significant.

## Results

### iPAECs from WT, GTKO, and TKO pigs proliferated stably and retained endothelial morphology

**Figure 1:**
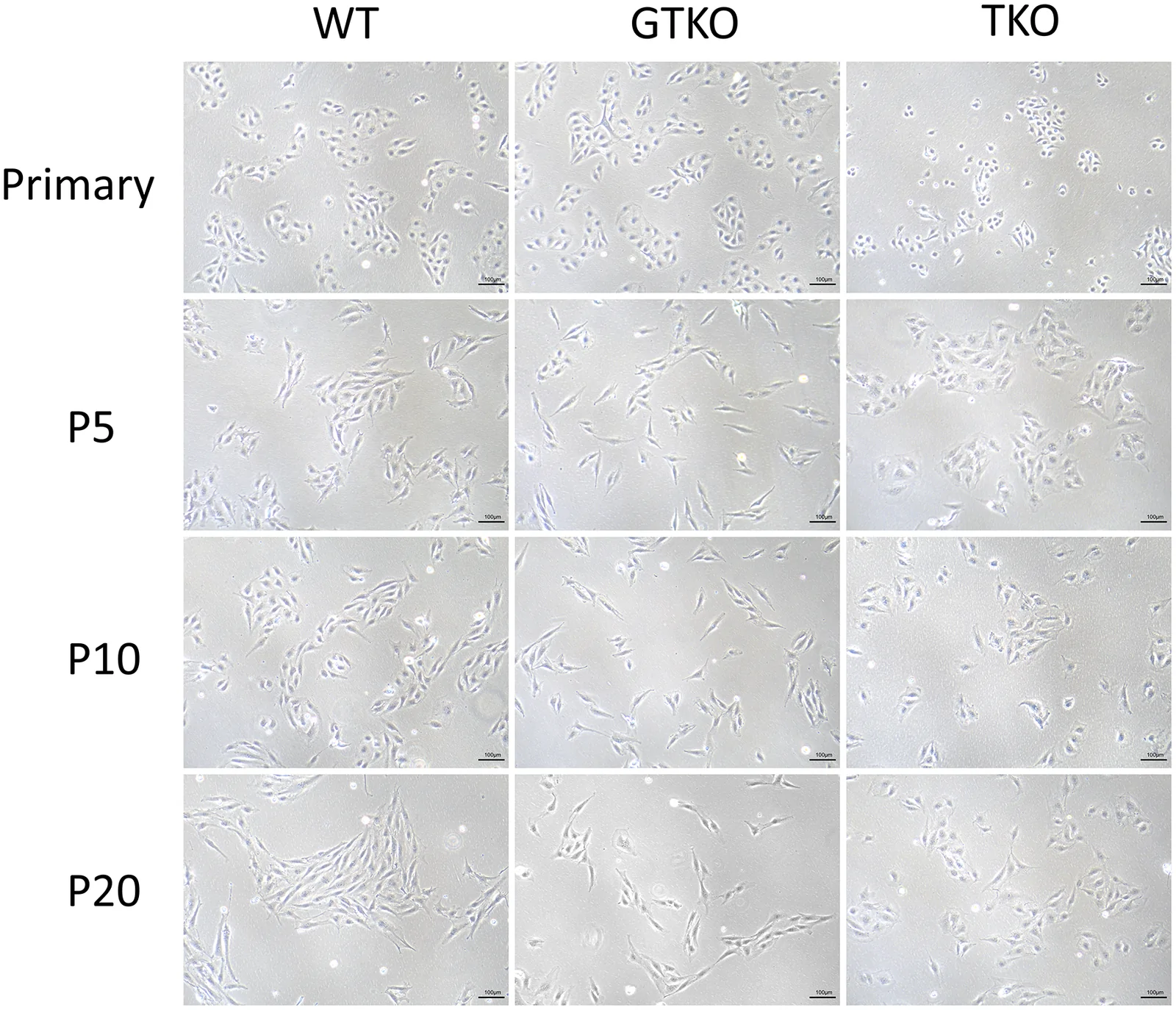
Morphology of WT, GTKO, and TKO pAECs and iPAECs across serial passages. Representative bright-field images showing the morphology of WT, GTKO, and TKO porcine aortic endothelial cells (pAECs) at the primary stage (Primary) and after immortalization at passages 5 (P5), 10 (P10), and 20 (P20). Scale bars, 100 μm.

To establish an *in vitro* model of porcine aortic endothelial cells representing different donor genotypes, primary aortic endothelial cells from WT, GTKO, and TKO donor pigs were immortalized. Their growth and morphology were monitored at the primary stage and across passages P5, P10, and P20 (**Figure 1**). Primary cells from all three genotypes attached well and displayed the typical “cobblestone” endothelial morphology, with distinct colony-like growth. After immortalization, WT, GTKO, and TKO cells were all stably propagated to P20, with no overt growth arrest or large-scale cell detachment observed during culture.

With increasing passage number, the immortalized cells of all three genotypes underwent varying degrees of morphological change, with some differences noted among the genotypes. GTKO iPAECs exhibited the most pronounced spindle-shaped transformation. WT iPAECs showed an intermediate degree of change, while TKO iPAECs retained more colony-like and cobblestone-like features even at the higher passages. Overall, immortalization primarily resulted in a shift from the typical cobblestone morphology toward varying degrees of spindle-shaped morphology. However, iPAECs of all three genotypes maintained stable proliferation across passages.

### iPAECs stably maintained their endothelial phenotype and genotype-matched xenoantigen profiles across multiple passages

**Figure 2:**
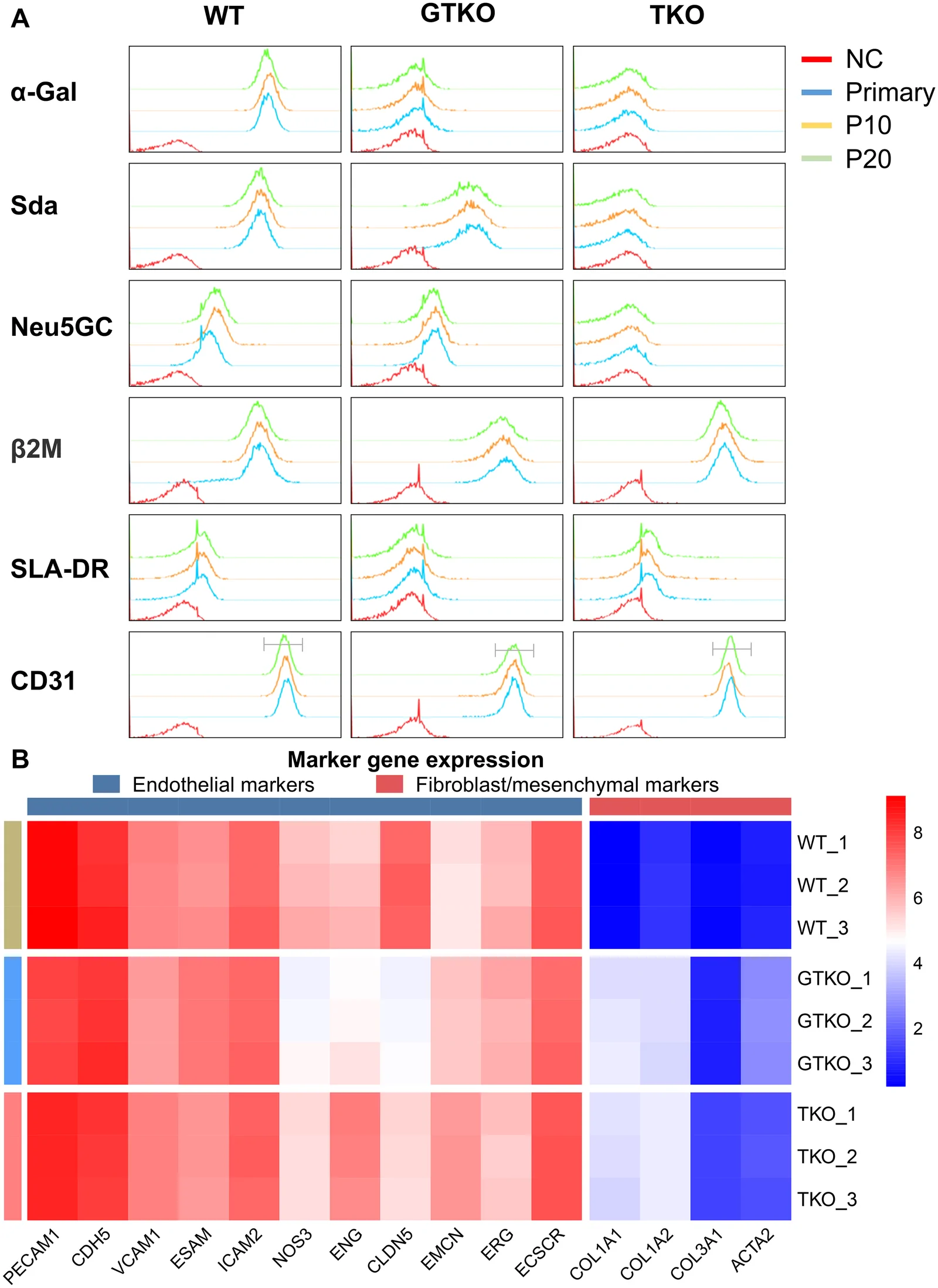
Phenotypic and transcriptional characterization of WT, GTKO, and TKO iPAECs. **(A)** Flow cytometric analysis of α-Gal, Sda, Neu5Gc, β2-microglobulin (β2M), SLA-DR, and CD31 expression on WT, GTKO, and TKO PAECs at the primary stage and after immortalization at passages 10 (P10) and 20 (P20). NC, negative control. **(B)** Heatmap showing the expression of endothelial-associated genes (PECAM1, CDH5, VCAM1, ESAM, ICAM2, NOS3, ENG, CLDN5, EMCN, ERG, ECSCR) and fibroblast/mesenchymal-associated genes (COL1A1, COL1A2, COL3A1, ACTA2) in WT, GTKO, and TKO iPAECs. WT_1–3, GTKO_1–3, and TKO_1–3 represent samples collected at passages P7, P12, and P16, respectively.

To determine whether the immortalized cells remained suitable for xenoimmunology research, we examined the stability of the endothelial phenotype and xenoantigen expression of the three iPAEC genotypes across serial passages. Flow cytometry demonstrated that WT cells continuously expressed α-Gal, Sda, and Neu5Gc at the primary stage and at P10 and P20. The GTKO cells lacked α-Gal but retained Sda expression, and TKO cells exhibited markedly reduced expression of α-Gal, Sda, and Neu5Gc (**Figure 2A**). These antigen expression patterns were consistent with the respective gene-editing backgrounds of the donor pigs and remained relatively stable up to P20. Additional analysis showed that the SLA class I-associated molecule β2M was consistently detected in all three iPAEC genotypes. However, baseline expression of the SLA class II molecule SLA-DR remained low to almost negligible in all three cell types, with no obvious change between the primary stage and P10 or P20. Furthermore, CD31 was consistently expressed at high levels (>95% positivity) across all three iPAECs, indicating that the immortalized cells retained a basic endothelial phenotype.

Transcriptomic analysis further showed that all three iPAEC genotypes continuously expressed a range of endothelial-associated genes, including PECAM1, CDH5, VCAM1, ESAM, ICAM2, CLDN5, EMCN, and ECSCR, at P7, P12, and P16 (**Figure 2B**). In contrast, fibroblast/mesenchymal-associated genes such as COL1A1, COL1A2, COL3A1, and ACTA2 were expressed at consistently low levels. Taken together, these iPAECs showed no obvious shift toward a fibroblast-like phenotype during serial passaging and stably maintained a xenoantigen expression profile consistent with their respective donor gene-editing backgrounds. Therefore, these cell types fulfilled the basic requirements for use in *in vitro* xenoimmunology research.

### Natural anti-pig antibody levels in human and rhesus monkey sera exhibited marked inter-individual variation when assessed using iPAECs

**Figure 3:**
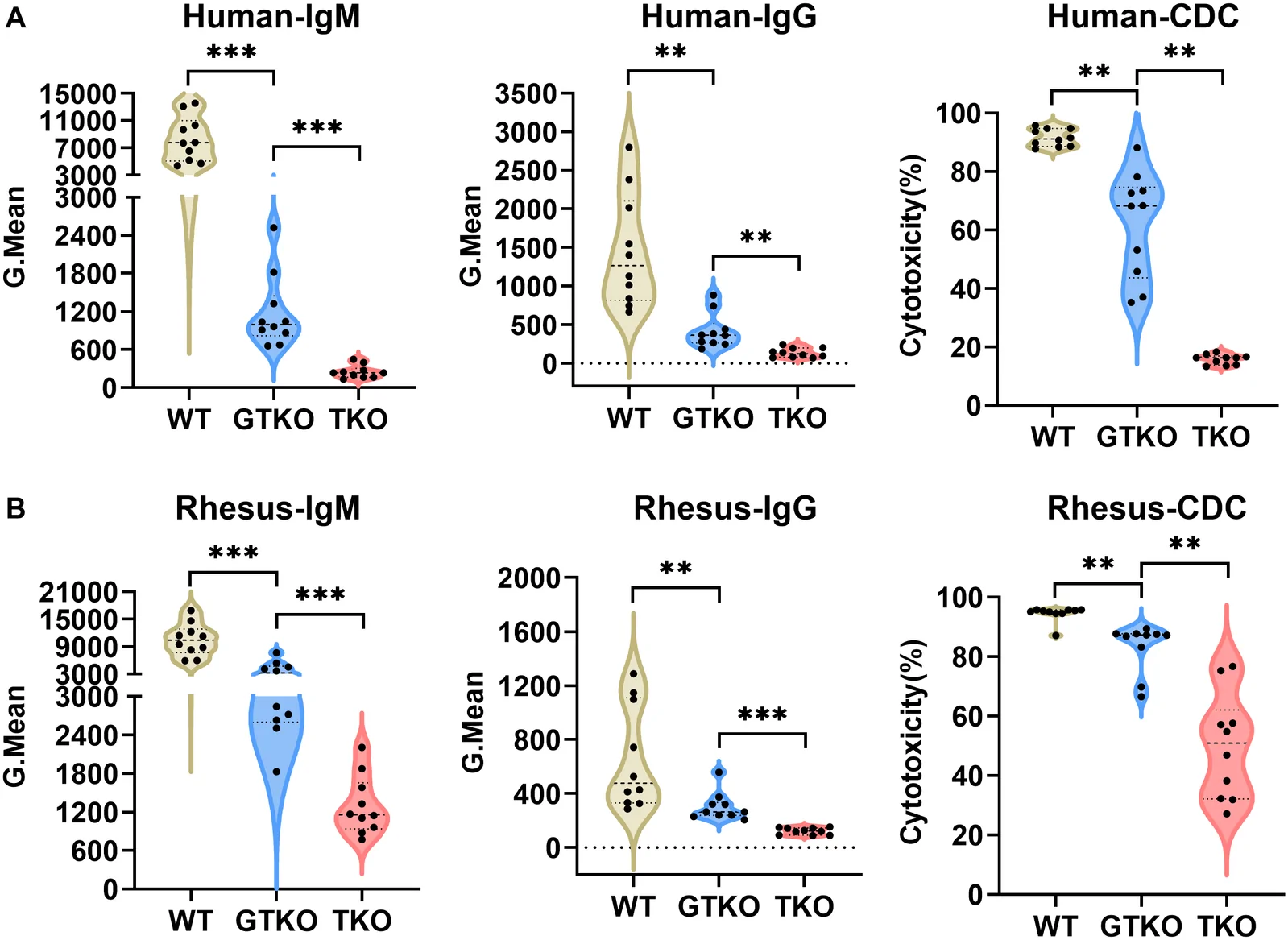
Detection of natural anti-pig antibodies in the sera of humans and rhesus monkeys using WT, GTKO, and TKO iPAECs. Ten human serum samples and ten rhesus monkey serum samples that had not been immunologically screened were randomly selected. **(A)** Anti-pig IgM/IgG binding and complement-dependent cytotoxicity (CDC) in human serum samples. **(B)** Anti-pig IgM/IgG binding and CDC in rhesus monkey serum samples. Anti-pig IgM/IgG binding and CDC were measured by flow cytometry using WT, GTKO, and TKO iPAECs as target cells. The results are reported as G.Mean. Data are presented as means ± SD.

To assess the potential utility of iPAECs as a screening tool for identifying low-antibody recipients, we determined whether iPAECs retained their ability to discriminate inter-individual differences in preformed natural anti-pig antibodies. Specifically, we assessed IgM and IgG binding and CDC against WT, GTKO, and TKO iPAECs using 10 human and 10 rhesus monkey serum samples. When WT iPAECs were used as target cells, human and rhesus monkey sera exhibited overall high levels of IgM and IgG binding, but substantial inter-individual variation was observed (**Figure 3**). The standard deviations (SD) for the IgM and IgG binding were 3303 and 730 for human sera and 3535 and 383.1 for rhesus monkey sera, respectively. These results demonstrated considerable variability in preformed natural anti-pig antibody levels across recipients. Despite this variation, CDC against WT iPAECs remained consistently high, with most samples exceeding 90%.

In GTKO iPAECs, antibody binding and CDC mediated by human and rhesus monkey sera were significantly reduced compared with the WT group (*p < 0.05*), although inter-individual differences persisted. In TKO iPAECs, antibody binding and CDC of human sera were further reduced (*p < 0.05*) and remained at low levels with relatively diminished inter-individual variation. In contrast, rhesus monkey sera retained comparatively high antibody binding and CDC against TKO iPAECs, with IgM and CDC values still showing pronounced inter-individual variability. Due to the observed inter-individual differences, all subsequent experiments were performed using pooled sera prepared from 20 human and 20 rhesus monkey serum samples to minimize the impact of inter-individual variability on experimental outcomes.

### iPAECs and PBMCs showed good consistency in detecting differences in preformed anti-pig antibody levels among rhesus monkeys

**Figure 4:**
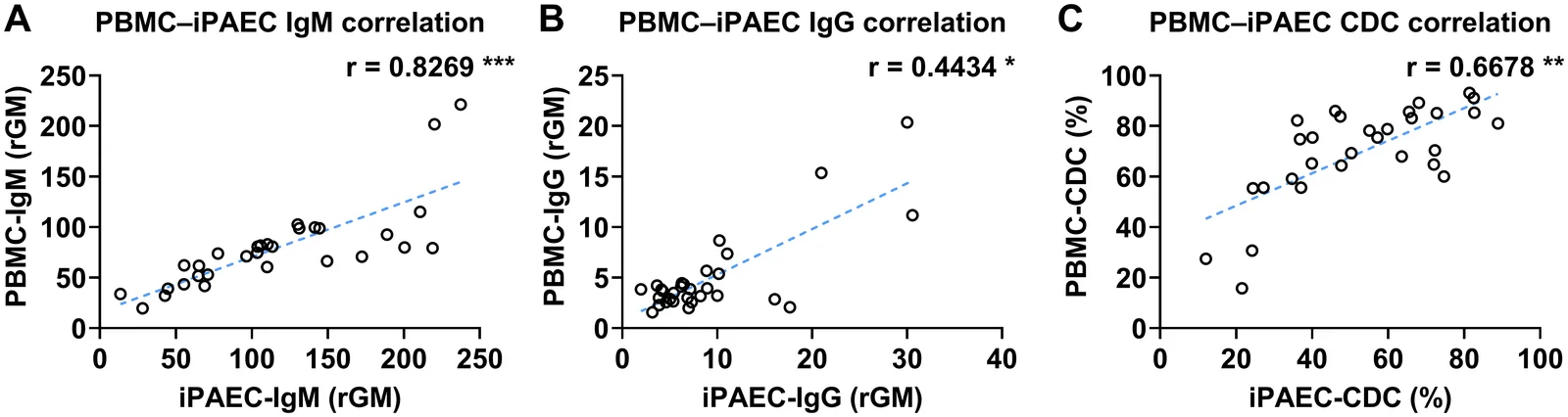
Correlations between IgM and IgG binding and CDC to TKO iPAECs and TKO PBMCs in rhesus monkey sera. IgM/IgG antibody binding and CDC were measured by flow cytometry after incubation of individual rhesus monkey serum (n = 30) samples with TKO iPAECs or TKO PBMCs. Correlations between TKO iPAEC- and TKO PBMC-based assays were analyzed for **(A)** IgM binding, **(B)** IgG binding, and **(C)** CDC. IgM and IgG binding were assessed as the relative geometric mean (rGM), calculated by dividing the geometric mean fluorescence for each sample by that of the negative control. Note the different scales of rGM and CDC on the x- and y-axes. r indicates the correlation coefficient.

To further assess whether iPAECs could serve as a surrogate target cell to identify low-preformed-antibody recipients in pig-to-rhesus monkey crossmatch screening, we compared results obtained from the same panel of rhesus monkey sera using two target cell systems, TKO donor-derived PBMCs and iPAECs. IgM and IgG binding levels and CDC were measured against both target cell types, and Spearman’s rank correlation was performed. Overall, results from the two systems were significantly and positively correlated. IgM binding showed the strongest correlation (r = 0.8269, *p <* 0.0001), followed by CDC (r = 0.6678, *p < 0.01*), while IgG binding demonstrated a weaker but still statistically significant correlation (r = 0.4434, *p < 0.05*) (**Figure 4**). These findings revealed that individuals exhibiting low or high antibody reactivity in the PBMC-based assay tended to show comparable trends in the iPAEC-based system. These observations suggested that iPAECs could accurately reflect inter-individual variation in preformed antibody responses among rhesus monkeys and might serve as an effective tool for *in vitro* pre-screening of low-antibody recipients.

### iPAECs stably reflected genotype-dependent humoral immune responses across serial passages

**Figure 5:**
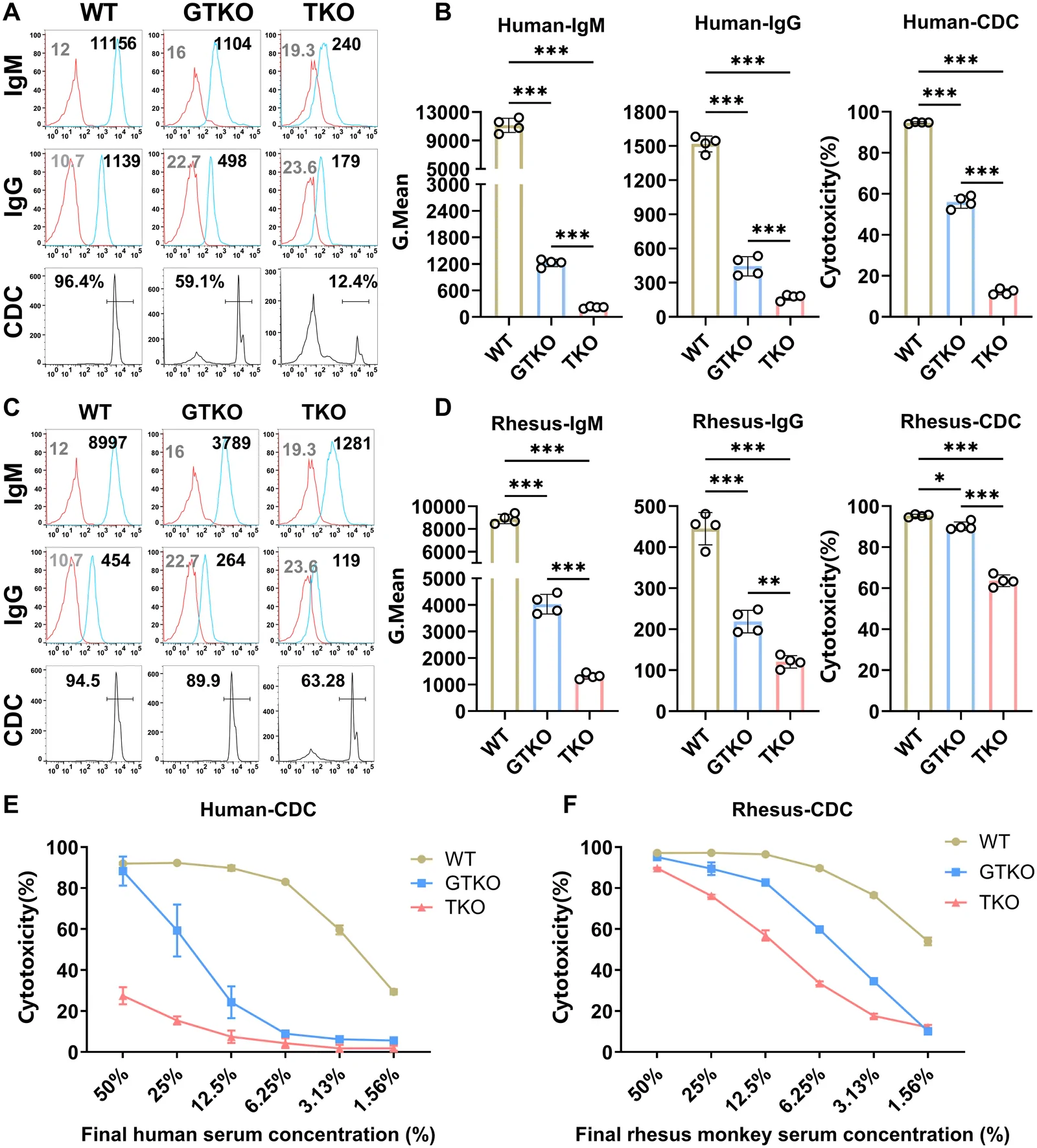
Comparison of human and rhesus monkey serum IgM/IgG binding and CDC against WT, GTKO, and TKO iPAECs. **(A, C)** Flow cytometric histograms showing representative binding of pooled human **(A)** or rhesus monkey **(C)** IgM/IgG antibody (top/middle row, serum diluted 1:10, **(G)** Mean values are shown) to pig iPAECs and CDC (bottom row, serum diluted 1:6, percentages are shown) against the same iPAECs. **(B, D)** Statistical analysis of pooled human **(B)** or rhesus monkey **(D)** serum antibody binding (IgM and IgG) to pig iPAECs and CDC against the same iPAECs. **(E, F)** Comparison of mean serum CDC (at serum concentrations of 50% to 1.57%) of pooled human **(E)** or rhesus monkey **(F)** against WT, GTKO, or TKO iPAECs. Each sample was measured three times. All data are presented as means ± SD (*P<0.05, **P< 0.01, ***P< 0.001; ns, non-significant). The P-value was obtained from three independent experiments.

To further evaluate the value of iPAECs of different genotypes in humoral immunology research, pooled human serum and pooled rhesus monkey serum were each incubated with WT, GTKO, and TKO iPAECs, and IgM/IgG binding levels and CDC were assessed. WT iPAECs showed high IgM and IgG binding and high CDC with human and rhesus monkey serum (**Figure 5**). Specifically, with human serum, the IgM G. Mean was approximately 11, 000 and the IgG G. Mean was approximately 1, 100, with CDC >90%. With rhesus monkey serum, the IgM G. Mean was approximately 9, 000 and the IgG G. Mean approximately 450, with CDC also >90%. These results indicated that WT iPAECs were strongly immunogenic to human and rhesus monkey serum. Compared with the WT group, GTKO iPAECs showed significantly reduced binding of IgM and IgG to both human and rhesus monkey serum (*p < 0.01*). Specifically, IgM/IgG binding and CDC with human serum were markedly reduced for GTKO iPAECs. In contrast, with rhesus monkey serum, although the values tended to be lower than in the WT group, they remained relatively high overall and could still mediate substantial CDC at higher serum concentrations.

Following knockout of Sda and Neu5Gc on the GTKO background, IgM/IgG binding and CDC with human serum were again significantly reduced for TKO iPAECs (*p < 0.01*), and CDC mediated by human serum remained below 20% across a serum concentration range of 1.56%–25%. In contrast, IgM/IgG binding and CDC with rhesus monkey serum were also somewhat reduced for TKO iPAECs compared with the GTKO group but remained relatively high overall (IgM G. Mean: ~1, 200; IgG G. Mean: ~120; CDC ~60% at a 1:6 serum dilution). Furthermore, they retained relatively high reactivity even after additional dilution. These results revealed that iPAECs stably reflect humoral immunological differences determined by the xenoantigen background of different donor genotypes. Loss of α-Gal alone markedly reduced antibody binding and CDC, and this reduction was further amplified by additional loss of Sda and Neu5Gc. This trend was largely consistent with our previous results obtained using PBMCs (26), indicating that the influence of different porcine xenoantigens on the humoral immune responses of humans and rhesus monkeys was similarly reflected by iPAECs.

**Figure 6:**
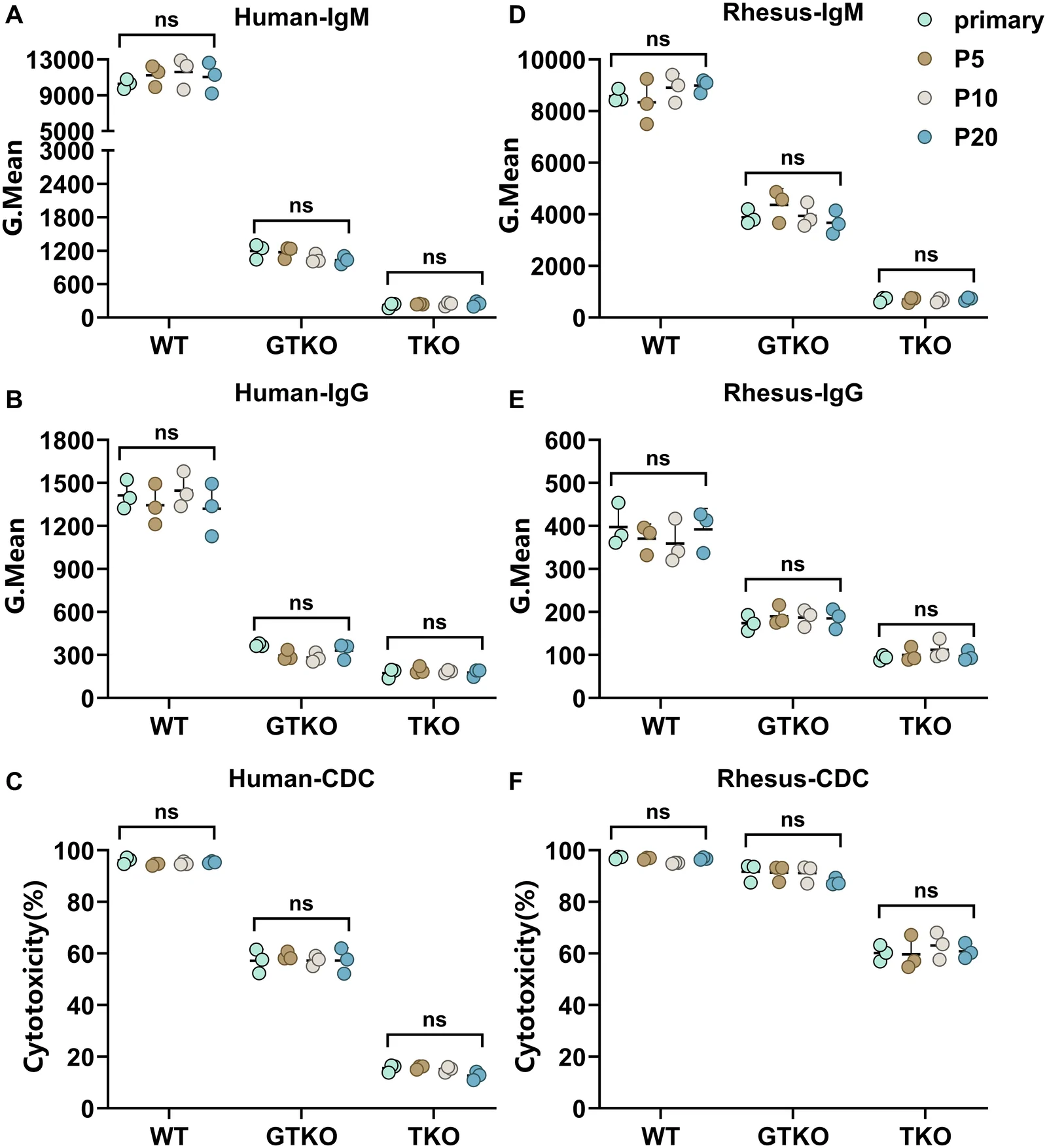
Evaluation of the stability of human and rhesus monkey serum IgM/IgG binding and CDC against WT, GTKO, and TKO iPAECs across serial passages. **(A–C)** Human serum IgM binding **(A)**, IgG binding **(B)**, and CDC **(C)** against WT, GTKO, and TKO iPAECs at the primary stage and passages 5 (P5), 10 (P10), and 20 (P20). **(D–F)** Rhesus monkey serum IgM binding **(D)**, IgG binding **(E)**, and CDC **(F)** against WT, GTKO, and TKO iPAECs at the primary stage and passages 5 (P5), 10 (P10), and 20 (P20). IgM and IgG binding are presented as geometric mean fluorescence intensity (G. Mean). Each dot represents an individual experiment. Data are presented as means ± SEM. (ns=not significant). The P-value was obtained from three independent experiments.

To further evaluate passage stability, we compared IgM/IgG binding levels and CDC after incubation with pooled serum for primary cells and for cells at P5, P10, and P20. Within each genotype, none of the indices differed significantly between passages (all ns, **Figure 6**), and the overall trends across WT, GTKO, and TKO iPAECs were consistent with the results described above, indicating that this system could be reliably used for humoral immunology research across serial passages.

### iPAECs could serve as stimulator cells to elicit robust human anti-pig T-cell immune responses in a xenogeneic MLR system

**Figure 7:**
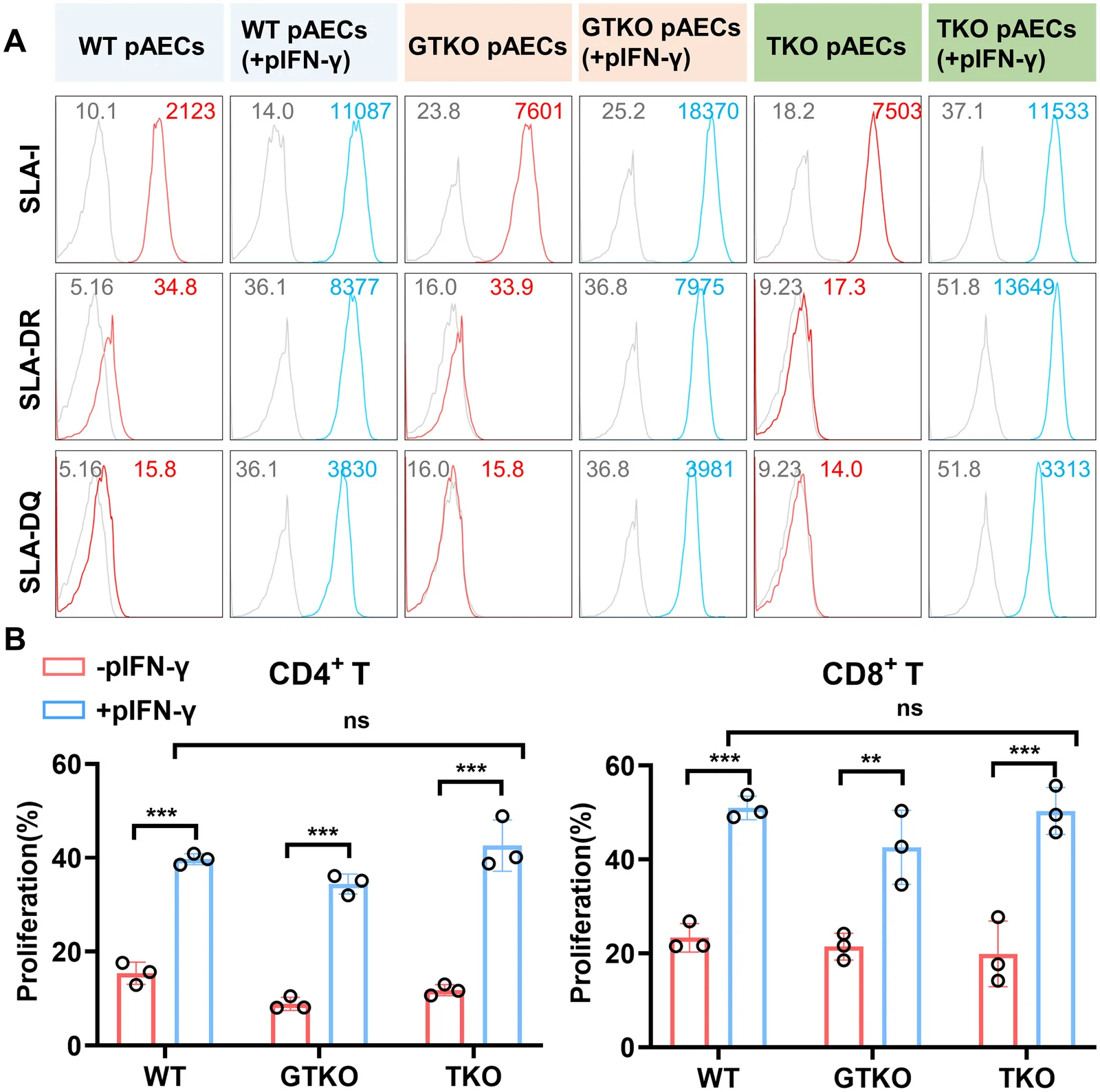
Human CD4+/CD8+ T‐cell proliferation in response to WT, GTKO, and TKO iPAECs. Xenogeneic mixed lymphocyte reactions (MLRs) were performed by co-culturing CFSE-labeled human PBMCs with irradiated WT, GTKO, and TKO iPAECs, either unstimulated or stimulated with pIFN-γ. After six days, the cells were harvested and labeled with anti-human CD3, CD4, and CD8 antibodies. **(A)** Expression of SLA-I and SLA-II (SLA-DR, SLA-DQ) on WT, GTKO, and TKO iPAECs with or without pIFN-γ stimulation (48 h), shown as geometric mean fluorescence intensity (G. Mean). Gray histograms represent isotype control staining, which were unstained cells for lectin experiments. **(B)** Summary data of human CD4^+^ and CD8^+^ T-cell proliferation in response to irradiated WT, GTKO, and TKO iPAECs with (blue) or without (red) pIFN-γ stimulation. Human T-cell proliferation was assessed using a CFSE dilution assay, and the percentages of dividing T cells beyond the parental generation are shown. Data are presented as the mean % proliferating cells ± SEM from at least three independent experiments (*p<0.05, **p<0.01, ***p<0.001, ns=not significant).

In addition to the humoral immunology studies, we evaluated the feasibility of using iPAECs as stimulator cells for xenogeneic T-cell immunology research. After treatment of WT, GTKO, and TKO iPAECs with pIFN-γ, expression of SLA-I, SLA-DR, and SLA-DQ was assessed, along with their capacity to induce human CD4^+^ and CD8^+^ T cell proliferation. pIFN-γ stimulation markedly upregulated SLA-I, SLA-DR, and SLA-DQ expression on iPAECs of all three genotypes (**Figure 7A**). Subsequently, irradiated, inactivated iPAECs were co-cultured with human PBMCs to establish a xenogeneic MLR system. iPAECs without pIFN-γ treatment induced measurable human T-cell proliferation, with CD4^+^ T-cell proliferation rates of approximately 10% - 20% and CD8^+^ T-cell proliferation rates of approximately 20%, with no significant differences among the three genotypes.

After pIFN-γ treatment, CD4^+^ and CD8^+^ T-cell proliferation induced by WT, GTKO, and TKO iPAECs was further enhanced (*p < 0.01*). The CD4^+^ T-cell proliferation rates increased to approximately 35% - 45%, and the CD8^+^ T-cell proliferation rates increased to approximately 35% - 55%, again with no significant overall difference among the three groups. These results indicated that iPAECs could serve as target cells for humoral immunology studies and as stimulator cells in a xenogeneic MLR system to assess human anti-pig T-cell immune responses. These results support the broad applicability of iPAECs for use in *in vitro* studies.

## Discussion

The porcine vascular endothelium is the first tissue encountered by recipient blood after reperfusion in organ xenotransplantation. Furthermore, porcine endothelial cells are the primary target of preformed anti-pig antibody binding, complement activation, and AMR (12). Thus, porcine endothelial cells are widely used to study the interactions between the recipient immune system and donor vascular endothelium *in vitro*. Commonly used porcine endothelial cells include porcine aortic endothelial cells, porcine renal microvascular endothelial cells, and porcine liver-derived endothelial cells, among others (16, 17, 24), with PAECs being the most widely used due to their relative ease of isolation and culture (12, 18, 22, 24). Previous studies have used PAECs primarily for the following purposes: 1) identifying novel xenoantigens (19); 2) serving as a complement to PBMC-based models in flow crossmatch and CDC assays to evaluate the risk of IgM/IgG binding and complement-mediated injury of porcine endothelial cells by human or NHP serum (18); 3) comparing differences in the binding of human or NHP natural anti-pig antibodies to porcine endothelial cells with different gene-editing backgrounds (8); 4) studying interactions between innate immune effector cells and porcine endothelium, including NK cell degranulation, antibody-dependent cellular cytotoxicity (ADCC), macrophage phagocytosis, and other endothelium-related innate immune injury mechanisms (21, 27); and 5) serving as stimulator cells in a xenogeneic mixed lymphocyte-endothelial cell reaction (MLR) system to assess human anti-pig T-cell immune responses (23, 28, 29).

However, primary PAECs have limited proliferative capacity *in vitro*, and phenotypic drift and functional instability may occur during multiple passages, which is undesirable for long-term, repeated, and standardized comparative studies. Therefore, we obtained primary PAECs from WT, GTKO, and TKO donor pigs and generated corresponding iPAECs by introducing the SV40 large T antigen. The SV40 large T antigen enhances *in vitro* cellular proliferative capacity by interfering with cell-cycle regulatory pathways such as p53 and Rb, delaying senescence, and suppressing growth arrest (17). The resulting iPAECs maintained stable, high CD31 and SLA-I expression across numerous serial passages, with consistently high cell purity. Moreover, iPAECs derived from donor pigs of different genotypes retained xenoantigen expression profiles (α-Gal, Sda, and Neu5Gc) consistent with their respective primary cells across serial passages. No loss of the relevant antigen expression was observed by passage 20. Notably, the WT, GTKO, and TKO iPAECs used in this study were each derived from a different donor pig, rather than generated through secondary gene editing of a single *in vitro* cell line. This strategy maximally preserved an antigenic background highly consistent with that of the donor pigs.

Although porcine vascular endothelial cells and porcine PBMCs share some porcine-derived antigens (such as α-Gal, Sda, and Neu5Gc), their antigen expression profiles are not identical. Previous studies have revealed that PAECs can be used to identify non-Gal endothelium-associated xenoantigens (19). A study of pig-to-human renal xenotransplantation found that antibody responses detected using PAECs were markedly stronger than those detected using PBMCs, and that changes in anti-PAEC IgG levels correlated more closely with AMR (12). Therefore, porcine endothelial cells can serve as a valuable complement to PBMCs for assessing donor vascular endothelium-related antibody responses and AMR risk.

In this study, we used human and rhesus monkey sera to further evaluate the immunogenicity of iPAECs derived from the three donor pig genotypes. The results showed that antibody binding and CDC levels against the three iPAEC genotypes varied markedly among individual human and rhesus monkey serum samples. Both human and rhesus monkey sera showed high IgM/IgG binding and CDC levels against WT iPAECs. After α-Gal knockout, antibody binding and CDC were significantly reduced. Following additional knockouts of Neu5Gc and Sda, antibody binding and CDC levels of human serum against TKO iPAECs remained low. However, rhesus monkey serum retained relatively high antibody binding and CDC responses against TKO iPAECs. These trends were broadly consistent with results obtained using PBMCs derived from pigs of the same genetic background (26).

We also performed flow crossmatch and CDC assays on 30 rhesus monkey serum samples using TKO iPAECs and TKO PBMCs and observed that the results from the two cell-based assays were significantly correlated. Together, these results indicated that the iPAECs established in this study retained the major xenoantigen features corresponding to their respective donor genotypes. Therefore, these cells can be used to evaluate humoral immune responses of human and rhesus monkey serum against porcine vascular endothelium and to screen for rhesus monkey recipients with low levels of preformed antibodies against TKO pigs. In addition, there is the potential to monitor changes in xenoreactive antibody levels after transplantation.

Passage stability is a key determinant of whether an immortalized cell line can be used for long-term, standardized research. Previous studies have demonstrated that immortalized porcine endothelial cells can retain their endothelial phenotype and xenoimmune response features to some extent (17, 24). These cells can also be used to assess IgM/IgG binding, complement activation, and CDC responses to human or NHP serum (18), as well as to compare the humoral immunogenicity of porcine cells with different gene-editing backgrounds (17). The iPAECs established in this study maintained stable antibody binding and CDC responses across serial passages and preserved the immune response differences among genotypes, indicating their major xenoantigen features did not change appreciably due to immortalization or passaging. Compared with primary PAECs, this system reduced experimental variability arising from differences in source batches and culture conditions, making it better suited for repeated, standardized assessment of the humoral immunogenicity of porcine endothelial cells with different gene-editing backgrounds.

Considering xenogeneic cellular immune responses, porcine endothelial cells differed markedly from human endothelial cells. Previous studies have revealed that human endothelial cells typically express little to no levels of the classical B7 costimulatory molecules, whereas porcine endothelial cells can express CD80/CD86 and promote human T-cell activation via the CD28 costimulatory pathway (23, 30–32). In addition, SLA-I on the surface of porcine endothelial cells, together with SLA-II upregulated by inflammatory stimulation, can participate in the direct recognition of porcine endothelial cells by human T cells, thereby giving porcine endothelial cells substantial capacity for antigen presentation and T-cell stimulation (28). Based on this information, we examined whether the iPAECs established in this study retained the T-cell stimulatory properties of primary porcine endothelial cells. The results showed that after pIFN-γ treatment, surface expression of SLA-I and SLA-II was significantly upregulated in WT, GTKO, and TKO iPAECs, accompanied by enhanced proliferation of human CD4^+^ and CD8^+^ T cells. Therefore, iPAECs retain the capacity to induce human T-cell responses after immortalization, with upregulation of SLA-II potentially playing a critical role in amplifying the response.

Meanwhile, T-cell proliferation levels did not differ significantly among the iPAEC genotypes, suggesting that knockout of the major carbohydrate xenoantigens does not dramatically reduce human anti-pig T-cell immune responses. This conclusion is consistent with that of Wang ZY et al. (33), who used porcine PBMCs as stimulator cells. Kim et al. similarly showed, in a study of pig-to-rhesus macaque renal xenotransplantation, that CD4^+^ T-cell depletion significantly prolonged graft survival (1). They also demonstrated that porcine stimulator cells expressing SLA-II induced stronger proliferation of rhesus macaque CD4^+^ T cells, and that this response was markedly attenuated when SLA-II was absent (1). Based on these published results as well as our own findings, it appears that iPAECs may be suitable for evaluating humoral immune responses, for serving as an *in vitro* model for studying human anti-pig T-cell responses, and for informing future SLA-targeted gene-editing strategies.

Certain limitations were noted in this study. The PAECs used were derived from the porcine aorta and therefore represent macrovascular endothelial cells. In contrast, AMR- and TMA-related immune injury after xenotransplantation occurs primarily in the microvascular bed of the transplanted organ, including the glomerular and peritubular capillaries in the kidney and the microvascular endothelium in the heart. Previous studies have suggested that endothelial cells from different vascular beds may differ in their xenoantigen profiles, which may influence their interactions with recipient immune components (17). Whether the immunogenic features reflected by the iPAECs in this study fully represent the microvascular endothelium of transplanted organs such as the kidney remains to be verified. Future work should consider establishing immortalized cell lines derived from microvascular endothelial cells of the porcine kidney, heart, and other organs to complement PAECs to more comprehensively assess the immunological features of endothelial cells from different vascular beds in xenotransplantation. In addition, each iPAEC line was derived from a single donor pig and therefore represents only one SLA background; the specific impact of individual SLA haplotypes on the T-cell response still needs to be clarified in future studies.

In summary, this study successfully established iPAECs derived from WT, GTKO, and TKO donor pigs. It demonstrated that they stably maintain an endothelial cell phenotype and major xenoantigen expression features across serial passages. These iPAECs also stably reflected differences in IgM/IgG binding levels and CDC responses between porcine endothelial cells of different genotypes and between human or rhesus monkey serum, and retained the capacity to induce human T-cell proliferation. Therefore, the iPAECs established in this study represent a reliable and reproducible *in vitro* cell model for evaluating humoral and cellular xenoimmune responses to porcine vascular endothelium with different donor gene-editing backgrounds.

## Data Availability

The original contributions presented in the study are included in the article/supplementary material. Further inquiries can be directed to the corresponding authors.
